# Lung Ultrasound and Caval Indices to Assess Volume Status in Maintenance Hemodialysis Patients

**DOI:** 10.24908/pocus.v8i1.15802

**Published:** 2023-04-26

**Authors:** Mujtaba Z Al-Saray, Ala Ali

**Affiliations:** 1 Nephrology and Renal Transplantation Centre, The Medical City Baghdad Iraq

**Keywords:** Hemodialysis, Iraq, Lung, POCUS, Volume

## Abstract

**Background: **Volume overload is common in end stage kidney disease (ESKD) and dialysis patients. Hence, the need for objective tools to detect such volume excess. Point of care ultrasound (POCUS) is a goal-directed, bedside examination to answer such a specific diagnostic question. **Methods: **One hundred Iraqi adult hemodialysis patients were recruited from February 1 to May 31, 2022. Primary clinical, dialysis data, and prescriptions were recorded. A nephrologist performed a POCUS examination after the last dialysis session of the week. In addition, an ultrasound examination of the chest was performed to detect B-lines and pleural effusion. Caval parameters included inferior vena cava (IVC) diameter and collapsibility index. **Results: **The mean age of the study group was 51.48 ± 14.6 years, with 53% males. The mean interdialytic weight gain was 2.74 ± 1.15 Kg. Lower limb edema and pleural effusion were present in 33% and 27%, respectively. Forty-seven percent of patients had >3 B-lines on lung ultrasound with a range of 12. Forty-three percent of patients had an IVC diameter of >2 cm, and 93% had <50% IVC collapsibility. In total, 97% of patients had evidence of excess volume by ultrasound criteria. IVC collapsibility index was the most prevalent sign of excess volume (93%). Patients without lower limb edema and pleural effusion had positive B-lines in 38.8% and 38.3%, an IVC diameter >2 cm in 46.2% and 38.3%, and IVC collapsibility <50% in 89.5% and 95.8% respectively. **Conclusion: **Iraqi maintenance hemodialysis patients are volume overloaded, which warrants proper intervention for detection and dialysis management. POCUS is a useful and easily performed technique to assess the volume status.

## Introduction

In maintenance hemodialysis (HD) patients, volume management is critical in the management plan. Volume overload is associated with hypertension, increased arterial stiffness, left ventricular hypertrophy, impaired oxygenation, cardiovascular morbidity, and mortality 1.

There are clinical and instrumental methods for assessing volume status, with strengths and limitations. Clinically, dialysis professionals examine body weight and blood pressure (BP). Neither weight changes nor blood pressure spikes provide an accurate assessment of volume status unless marked volume overload expresses itself on clinical ground 2. Laboratory biomarkers like brain natriuretic peptide (BNP) and pro-BNP are well known for their usefulness in heart failure. However, they are less useful for fluid overload because of limited specificity 3. The complexity of the bioelectrical impedance procedure limits its use for assessing volume status. Furthermore, it cannot differentiate between increased plasma volume and expanded extravascular volumes like ascites and pleural effusion 4. 

In recent years, the use of ultrasound for volume status determination has increased. Point of care Ultrasound (POCUS) has become a valuable tool for volume status determination 5. It has been integrated into diverse areas of clinical practice since the early 1990s, and there has been renewed interest in POCUS as an adjunct to physical examination 6. Nephrology has many applications for POCUS. These include but are not limited to vascular access insertion and management, renal bedside ultrasound, and volume assessment of dialysis patients. In addition, nephrology fellowship programs are increasingly adopting nephrology-specific POCUS applications as part of their training curricula 7, 8.

Lung POCUS is a non-invasive and radiation-free procedure that can estimate extravascular lung water (EVLW), detect pulmonary congestion before it is clinically evident, and aid in dry weight determination. Lung B-lines are hyperechoic reverberation artifacts between the edematous septa and they reflect EVLW. In addition, pleural effusion can be easily detected 9. The LUST study (The Agreement between Auscultation and Lung Ultrasound in Hemodialysis Patients) demonstrated the better performance of ultrasound-detected B-lines in reflecting EVLW than pulmonary crackles and pulmonary edema 10.

Other markers of excess volume, such as inferior vena cava (IVC diameter ≥ 2 cm) and collapsibility (<50%), can also be detected by POCUS and help estimate central venous pressure and help the clinician in determining a dry weight. Combining lung B-lines and IVC parameters can provide objective evidence for volume status even before florid signs and symptoms appear. By assessing IVC parameters, Brennan JM et al. reported that 10% of the patients who left HD at or below goal weight were still hypervolemic 11, 12, 13.

There are approximately 8000 HD patients in Iraq, and, as in most developing countries, most patients are under-dialyzed. A previous Iraqi report described the applicability and utility of bioelectrical impedance in assessing the volume status of maintenance HD patients. Unfortunately, the application of bioimpedance in Iraqi HD units is still minimal 14.

In this study, we describe the first-time application of lung and IVC POCUS protocol to an Iraqi outpatient HD unit to assess volume status.

## Patients and Methods

Study design: An observational cross-sectional study

Setting: The study was conducted at the outpatient hemodialysis unit of the Medical City teaching hospital, Baghdad, from February 1 to May 31, 2022.

Patients: Adult ESKD patients on maintenance HD for >3 months were enrolled. Patients with a previous or current diagnosis of left ventricular dysfunction documented clinically and an ejection fraction of <50% were excluded. Patients with interstitial lung disease, cancer, active infection, debilitating illness, or an intercurrent acute disease were excluded. 

Sample size: With a 10% global prevalence of CKD, we used the following equation to calculate the sample size 15, 16: N = (z)2× (p × q)/d2= (1.96)2× (0.10 × 0.89)/(0.05)^2^

Where: 

N = sample size,

Z = standard deviation at a level of confidence 95% (1.96),

P = Population proportion (estimated frequency of occurrence of CKD, 10%),

[q = 1– p = the frequency of nonoccurrence, and

d = degree of precision (5%). 

Morning shift patients were examined on the last two days of the unit's weekly program to ensure examining them after the last HD session, specifically within 2 hours of completion of the session. Therefore, each patient was examined uniquely. Based on the sample size calculation and application of exclusion criteria, we initially recruited 127 patients. However, we could not have a clear ultrasound view in twenty-one patients, and six patients had incomplete data, so we excluded them from the study. 

Data collection: All patients' basic clinical and laboratory data and dialysis prescriptions were reviewed and recorded from their records. Pre- and post-dialysis clinical volume assessment was done after the last dialysis session of the week. The clinical evaluation checked for the presence/absence of dyspnea, peripheral edema, urine output, blood pressure, and pulmonary crackles on auscultation. In addition, body weight, dry weight, and interdialytic weight gain were measured and recorded. Dry weight is the lowest post-dialysis weight achieved after ultrafiltration without symptomatic hypovolemia 17.

For clinical edema, the following scale was used: 1, no clinical edema; 2, slight pitting (2 mm depth) with no visible distortion; 3, somewhat deeper pit (4 mm) with no readily detectable distortion; 4, noticeably deep pit (6 mm) with the dependent extremity full and swollen; and 5, very deep pit (8 mm) with the dependent extremity grossly distorted 18.

For urine output, patients were categorized as group 1 (<500 cc/day), group 2 (500-1000 cc/day), and group 3 (>1000 cc/day).

Ultrasound examination: For the ultrasound examination, we used a Clarius HD scanner from Clarius Mobile Health, Canada, 2020. The scanner is connected by Bluetooth to an iOS 15 iPad and can provide images exported at different image extensions. 

The examination was performed by a nephrologist who underwent one-month training by radiology and software experts from Clarius Mobile Health in Iraq. Then, an initial pilot of 20 patients was completed under the experts' supervision before embarking on the study and data collection.

Volume assessment includes the examination of the lungs for B-Lines and pleural effusion and the subcostal examination for IVC diameter and collapsibility. 

**Figure 1:**
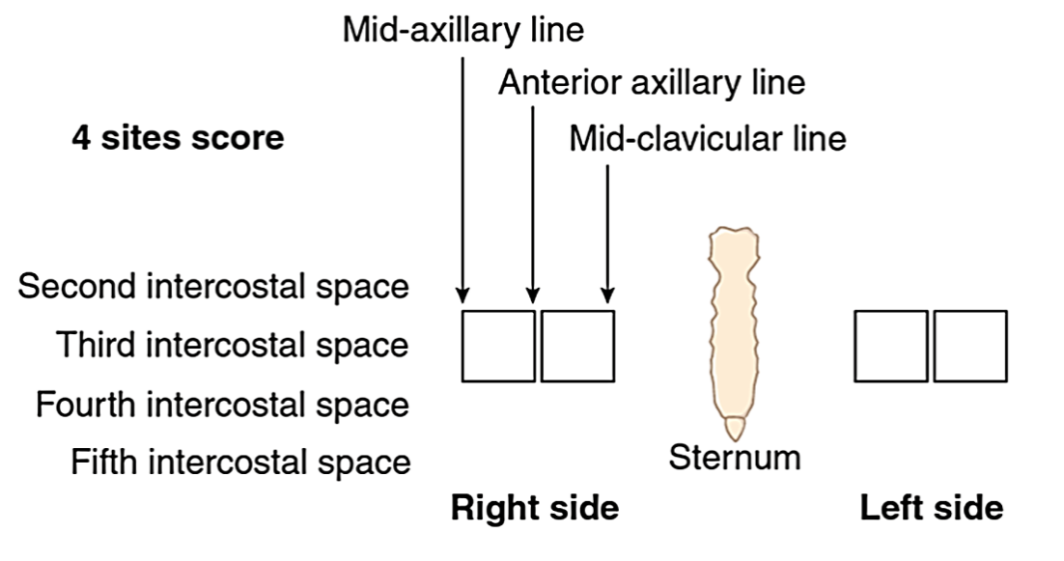
Four zones method and B-Lines on Lung ultrasound 19. Adapted with permission from Zoccali C,Mallamaci F, Picano E. Detecting and Treating Lung Congestion with Kidney Failure. Clin J Am Soc Nephrol. 2022 May;17(5):757-765. (Order License number 1293529-1).

Lung examination: Patients were placed in a supine position. Clarius C3 HD curvilinear probe was used in a lung setting with a 5 Mhz frequency. As previously described, a four-zone lung protocol was used as in Figure 1 19. Then, both lung bases were scanned for pleural effusion. 

**Figure 2:**
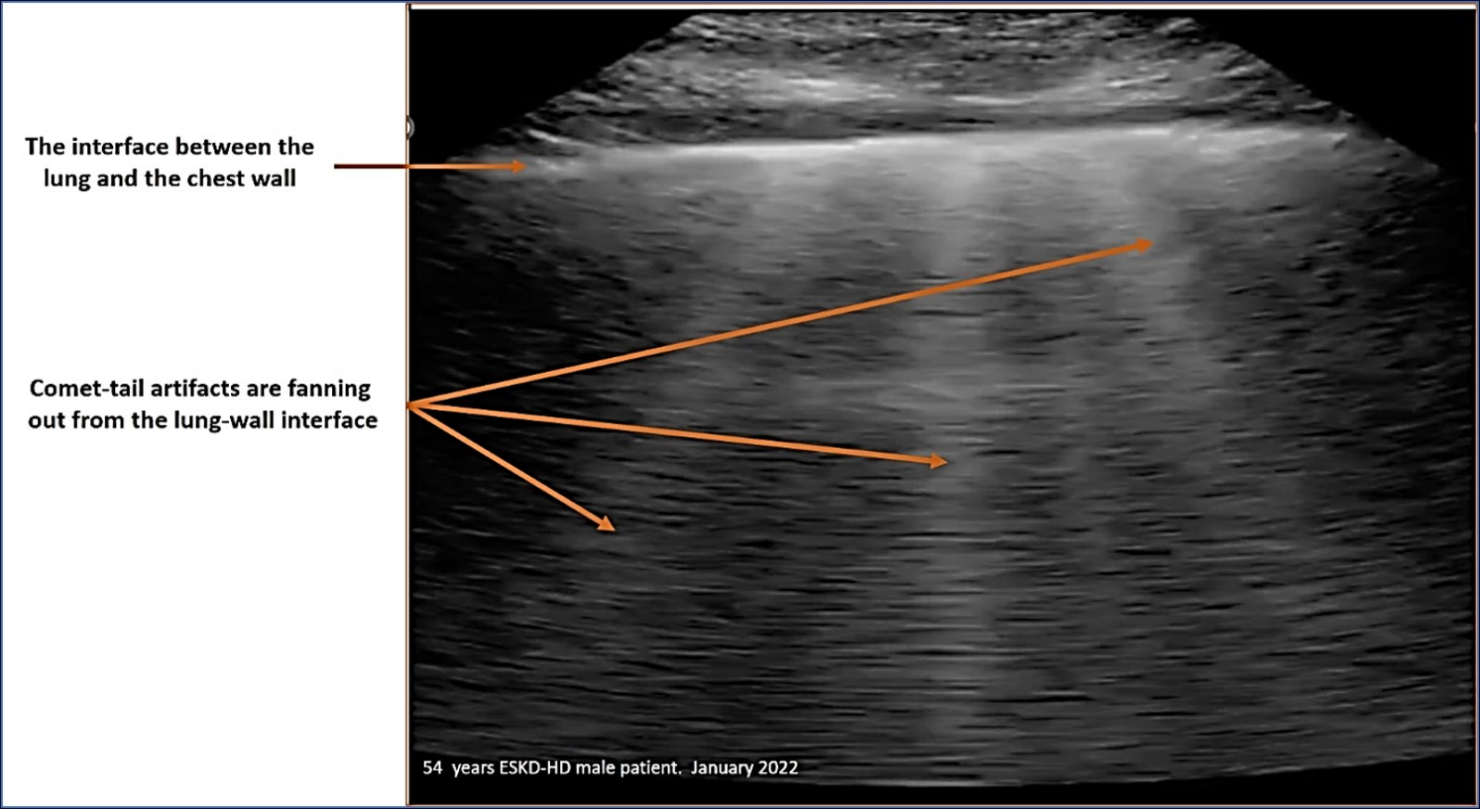
Examination of the Lung B-Line.

The B lines are hyperechoic, well-defined, comet tail artifacts arising from pleural line and extend indefinitely, erasing A-lines, moving with lung sliding if present. Two or fewer B-lines in each section are considered normal. The presence of more than three B-lines in at least one lung zone is consistent with the definition of EVLW. The more B-lines counted across the chest, the more pulmonary congestion correlates with EVLW. Thus, the number of B-lines in four areas was compiled and categorized into three groups (0-<3, ≥3-<10, and ≥ 10; Figure 2) 20. Visual assessment for the presence or absence of pleural effusions made at the bedside by placing the curvilinear probe in the midaxillary line 11, 21, 22.

**Figure 3:**
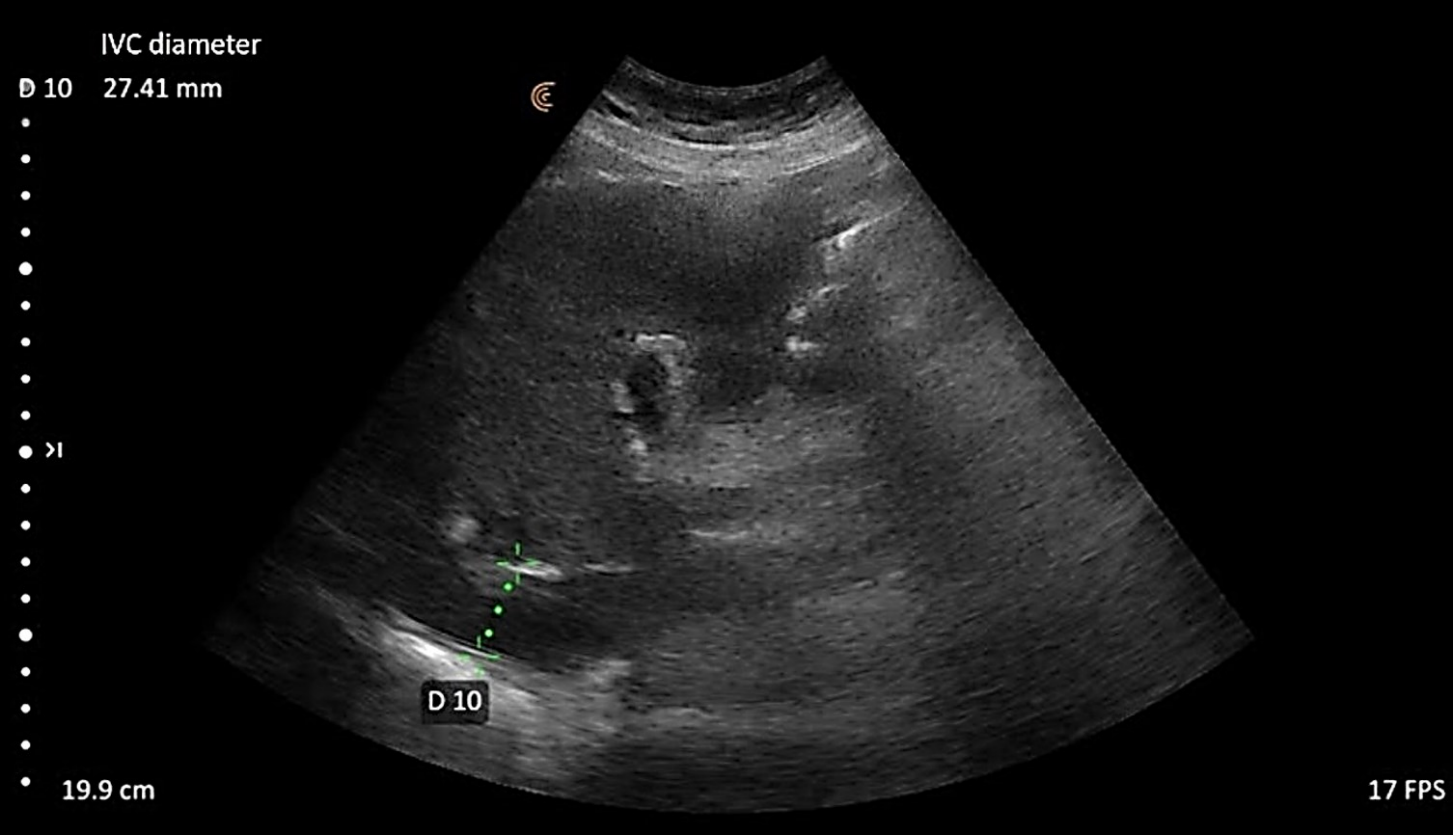
Measurement of IVC diameter on abdominal ultrasound.

IVC diameter and collapsibility: We used a curvilinear probe, 2.5 Mhz, in a cardiac setting. The IVC is viewed in the anterior sub-sternal window in the supine position, Figure 3. The IVC maximum diameter of more than 2 cm is hypervolemia 13.

Collapsibility index is calculated using the following formula 13:

Collapsibility index = ((IVC max –IVC mini /IVC max *100

Collapsibility index < 50 % is consistent with hypervolemia.

Ethical and regulatory issues: The study was approved by the Arab council of nephrology in Iraq. According to the 2018 Iraqi code of research ethics, all patients had consented to and approved the examination. In addition, all data retrieved from the records were recorded and ensured anonymity. 

Statistical Analysis: Statistical analyses were conducted with SPSS software (version 25.0, released in 2017; IBM SPSS Statistics for Windows). The descriptive statistics of the studied sample are presented as mean, standard deviations, frequency, and percentage. The ANOVA test was used to compare the means of more than two groups. In addition, we used the post hoc test (Bonferroni test) to discover differences among specific groups' mean values. Pearson's correlation was done to measure correlation among quantitative variables. P <0.05 was considered statistically significant. 

## Results

The mean age of the study group is 51.48 ± 14.6 years, with 53 males. Table 1 represents the baseline demographic characteristics of the study group. Only 20% had dialysis three times per week, and all participants had less than 4 hours of dialysis session time.

**Table 1:** Baseline characteristics of the study group.

Age _years _(Mean ± SD) 18 - ≤40 41 - ≤65 > 66	51.48 ± 14.6 24 67 9
Gender (Male/Female) ESKD etiology DM HT DM and HT GN Cystic disease Drugs/NSAID Unknown	53/47 - 30 16 16 11 4 8 15
HD Vintage _years _(Mean ± SD) ≤ 3 > 3 < 6 ≥ 6	2.9 ± 1.6 67 25 8
Dialysis Access AVF TDL	- 85 15
Dialysis Frequency 1/wk 2/wk 3/wk	- 5 75 20
Dialysis Duration _Hrs._ (Mean ± SD) 3 hours 3.5 hours	3.47 + 0.128 7 93
AVF, Arteriovenous Fistula; DM, Diabetes Mellitus; HT, Hypertension; GN, Glomerulonephritis, NSAID, Non-steroidal anti-inflammatory drug; TDL, tunneled dual lumen catheter;	

Table 2 describes the clinical and laboratory indicators of volume status. Fifty-one (51%) of the study group had a urine output of < 500 ml/24 hr. We did not include the jugular venous pulse in the analysis as it was fibrosed in 80% of the participants due to the previous dialysis catheters.

**Table 2:** Clinical and Laboratory indicators of volume status.

Urine Output _ml/24hr_ < 500 _ml_ 500 - < 1000 > 1000	- 51 5 44
Weight _Kg _(Mean ± SD)	77.82 ± 17.66
Dry weight _kg _(Mean ± SD)	74.57 ± 16.92
Interdialytic weight gain (IDWG) _Kg_ (Mean ± SD)	2.74 ± 1.15
Lower Limb edema G1 G2 G3 G4 G5	- 67 5 11 2 15
Systolic Blood Pressure _mm/Hg_ (Mean ± SD) SBP ≤ 110 SBP ≥ 120 < 150 SBP ≥ 150 < 180 SBP ≥ 180	151.7 ± 25.14 8 31 44 17
Diastolic Blood Pressure _mm/Hg_ (Mean ± SD) DBP < 80 DBP ≥ 80 < 100 DBP > 100	82.9 ± 12.73 34 46 20
Pleural Effusion	27
Hb _g/dl_ (Mean ± SD)	9.55 ± 1.36
Serum Na _Meq/l_ (Mean ± SD)	134.79 ± 1.36
Serum Albumin _g/dl_ (Mean ± SD) G1 ≤ 3 G2 >3 ≤ 4 G3 > 4	3.94 ± 0.72 11 42 47

Table 3 shows the parameters of Volume excess (VE) as measured by ultrasound; B-lines, IVC diameter, and IVC collapsibility. Forty-seven (47%) had more than 3 B-lines on lung ultrasound (Figure 2), and 43% had an IVC diameter of >2 cm. The most frequent feature of excess volume was IVC collapsibility of <50%, as it is detected in 93% of the study group. Thirty-nine patients (39%) had IVC > 2 cm and IVC CI <50%. (Figure 3).

**Table 3:** Ultrasound parameters of excess volume.

**B-Lines** Median Range G1 (0 - <3) G2 (≥3 - <10) G3 (≥10)	- 2 12 53 34 13
**IVC diameter** **_Cm_** **(Mean ± SD)** G1 <2 cm G2 >2 cm	1.95 ± 0.4 57 43
**IVC Collapsibility** **_%_** **(Mean ± SD)** G1 < 50% G2 > 50% **Number of patients with IVC d <2 cm and IVC CI < 50%**	21.03 ± 16.69 93 7 39

**Figure 4:**
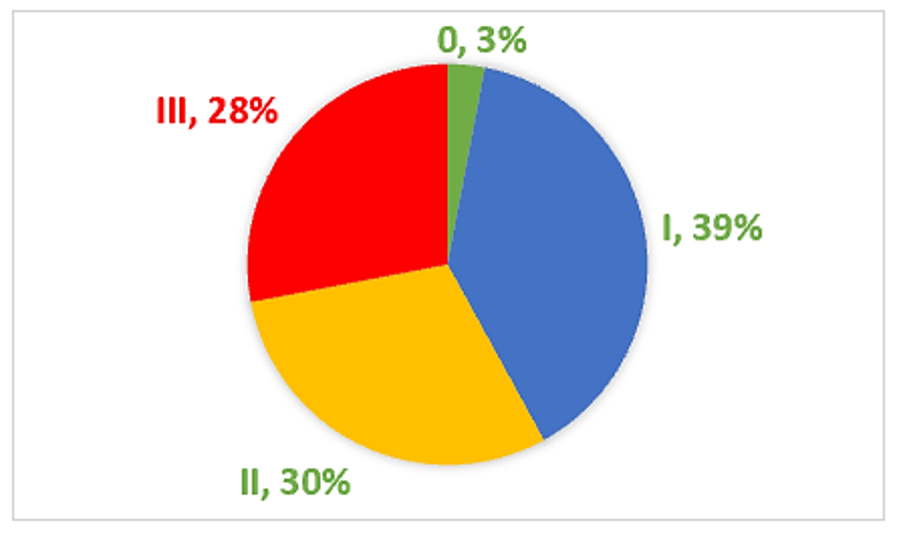
Patients with different ultrasound criteria of Volume Excess (VE). Thirty-nine patients (39%) had a single US criterion of VE, 30% had two criteria, and 28% had all three criteria, Lung B-lines, positive IVC diameter, and collapsibility index. Only 3% had no ultrasound evidence of VE.

Figure 4 shows the number of patients who fulfilled the ultrasound definitions of volume excess. While 97% showed 1 to 3 criteria of VE by ultrasound, only 3% had no ultrasound evidence of VE. Thirty-nine patients (39%) had a single US criterion of VE, 30% had two criteria, and 28% had all three criteria, Lung B-lines, positive IVC diameter, and collapsibility index. 

There were statistically significant associations between the three ultrasound criteria and the predialysis weight, dry weight, Systolic BP, serum sodium, and serum albumin (P values <0.01).

resents the correlation between thepredialysis parameters and the caval indices on ultrasound. Interestingly there is a negative correlation between serum albumin and the IVC indices. 

**Table 4:** Correlation betweenPredialysis parameters and caval indices.

	**SBP**	**DBP**	**Wt.**	**Dry Wt.**	**IDWG**	**Na**	**Albumin**
**IVC D**	.226*	.080	.040	.069	-.176	-.213	-.319**
**IVC IC**	-.457**	-.290**	-.162	-.131	-.056	-.142	-.248*
** Correlation is significant at 0.01 level * Correlation is significant at 0.05 level							

Table 5 shows that even in the absence of lower limb edema and pleural effusion, lung ultrasound and IVC study could still reveal incipient volume excess in up to 60% of the study group.

**Table 5:** Ultrasound findings in patients with Negative clinical signs of VE

**Clinical Sign**	**B-lines > 3**	**IVC d > 2 Cm + CI < 50%**
LL edema Negative	26/67 (38.8%)	39/67 (58.2%)
Pleural eff. Negative	28/73 (38.3%)	39/73 (53.4%)

## Discussion

Hemodialysis adequacy and volume control are the main pillars of dialysis prescriptions that would translate into severe clinical consequences. Unfortunately, volume management and dry weight measurement decisions are not always straightforward without objective tools that assess VE 23.

In this study, we described the easy application of POCUS in assessing the volume status of adult hemodialysis patients. Here, 97% of the study group showed variable degrees of VE by Ultrasound. 

According to the hemodialysis consensus, the study cohort received inadequate dialysis in frequency and the prescribed dialysis time. This will translate into inadequate volume control with 2.74 ± 1.15 kg interdialytic weight gain and high systolic and diastolic BP in >60%. Practices involving volume assessment and management protocols were associated with lower all-cause mortality (hazard ratio [HR], 0.78) 24, 25. 

In this study, a nephrologist performed a bedside ultrasound examination to answer a focused clinical question on volume status in this cohort. It is an increasing practice for nephrologists to apply POCUS because of ease and the short time to have proficiency. For example, the American college of chest physicians provides a 3-day training course for lung ultrasound. They have reported that novice learners can obtain proficiency after this course 26. A renal nurse can reliably perform an ultrasound of the IVC in hemodialysis patients and obtain high-quality scans for volume assessment 27. Being used for the first time in Iraq, our nephrologist had a one-month training by experts before the study conduction. 

The lung is the organ system more sensitive to the adverse effects of VE 23. The most widely validated protocol is 28-zone lung scanning, but it is a cumbersome procedure. It has been shown recently that 4-, 6-, or 8-zone lung ultrasounds were comparable to 28-zone studies for assessing pulmonary congestion 19, 21, 22. In an Egyptian study of 38 hemodialysis patients, the mean B-line score (BLS) was 10.32 ± 6.22, correlated with the volume status and guided the subsequent dialysis prescription 28. Pleural effusions are easily visualized with lung POCUS by placing a curvilinear probe in the midaxillary line 29. In our study, 27% of the participants had pleural effusions on ultrasound. 

In this study, the IVC collapsibility index (IVC CI) of <50% was the most prevalent sign of VE. The clinical value of the IVC CI has been documented in many clinical settings, like different types of shock, AKI, and to assess volume in chronic hemodialysis patients. Patients with lower IVC CI are more likely to tolerate ultrafiltration with hemodialysis 30.

The ultrasound signs of VE are significantly associated with predialysis clinical and laboratory parameters as the cohort is basically volume overloaded with high systolic blood pressure and dry weight. Both BLS and IVC parameters correlate with accumulated interdialytic weight 31. Furthermore, caval indices are significantly associated with all these parameters, as confirmed by the Pearson correlation study in Table 4. Hypoalbuminemia and low oncotic pressure contribute to the pathophysiology of VE and systemic venous congestion, and it is an important confounder that should be targeted. In the lung territory, hydrostatic pressure has a peculiar regulation 17. A larger volume may be needed to alter this regulation, and post-dialysis testing could explain the less significant association with B-lines on lung ultrasound. 

VE ultrasound patterns can be used to follow the patient’s status before and after ultrafiltration therapy. Unfortunately, there were no data about the ultrafiltration rate on patients' records. Thus, we could not calculate and analyze the rate. In only a few cases not included in the study protocol and analysis, we addressed some dialysis emergencies for hemodynamically unstable patients using POCUS, like pericardial effusion. 

In this study, chest ultrasound for B-lines and IVC parameters detected evidence of VE even in patients without evident limb edema or pleural effusion. Considering that the examination was performed after the last dialysis session of the week, this means incipient VE that had not been targeted by ultrafiltration. It was reported that 71% of patients with ESKD had imaging evidence of lung congestion and were utterly asymptomatic 1.

The LUST trial confirmed the utility of ultrasound over lung auscultation and peripheral edema 10. The latter two poorly reflected EVLW as measured by B-lines. Furthermore, Alexiadis et al. demonstrated the utility of lung ultrasound over other methods for evaluating dry weight and fluid status and helping recognize asymptomatic lung congestion (ROC 0.81-0.83) 32.

A more extensive study with a more representative sample will add more evidence. Despite an analytical element, the study’s cross-sectional design is a limitation. A study design that compares POCUS to clinical assessment and/or gold-standard tools is necessary. Yet, it is proof of concept of the utility of POCUS in volume assessment at hemodialysis facilities. 

In conclusion, Iraqi maintenance hemodialysis patients are volume overloaded, which warrants proper intervention for detection and management. POCUS is a useful and easily performed technique to assess the volume status. A more extensive study for evaluating the utility of POCUS in guiding volume management would be mandatory. Nephro-POCUS training should be part of nephrologist and renal nurses' training curricula. 

## Funding

The investigators received no grants or monetary funds during study conduction apart from self-fund. Clarius Mobile Health in Iraq provided the device without financial support or funds.

## Conflict of Interest Disclosure

None of the authors have any conflicts of interest to disclose
